# Selection Pressure Profile Suggests Species Criteria among Tick-Borne Orthoflaviviruses

**DOI:** 10.3390/v16101554

**Published:** 2024-09-30

**Authors:** Andrei A. Deviatkin, Yulia A. Aleshina, Galina G. Karganova, Alexander N. Lukashev

**Affiliations:** 1 Federal Research Center for Innovator and Emerging Biomedical and Pharmaceutical Technologies, 125315 Moscow, Russia; 2 Laboratory of Postgenomic Technologies, Izmerov Research Institute of Occupational Health, 105275 Moscow, Russia; 3 Martsinovsky Institute of Medical Parasitology, Tropical and Vector Borne Diseases, First Moscow State Medical University (Sechenov University), 119435 Moscow, Russia; vjulia94@gmail.com (Y.A.A.); alexander_lukashev@hotmail.com (A.N.L.); 4 Faculty of Bioengineering and Bioinformatics, Lomonosov Moscow State University, 119234 Moscow, Russia; 5 Federal State Autonomous Scientific Institution “Chumakov Federal Center for Research and Development of Immune-and-Biological Products of the Russian Academy of Sciences” (Institute of Poliomyelitis) (FSASI “Chumakov FSC R&D IBP RAS”), 108819 Moscow, Russia; karganova@bk.ru; 6 Department of Organization and Technology of Production of Immunobiological Preparations, Institute for Translational Medicine and Biotechnology, First Moscow State Medical University (Sechenov University), 119048 Moscow, Russia; 7 Research Institute for Systems Biology and Medicine, 117246 Moscow, Russia

**Keywords:** taxonomy, tick-borne encephalitis, Omsk hemorrhagic fever

## Abstract

Orthoflaviviruses are arthropod-borne viruses that are transmitted by mosquitoes or ticks and cause a range of significant human diseases. Among the most important tick-borne orthoflaviviruses (TBFVs) is tick-borne encephalitis virus (TBEV), which is endemic in Eurasia, and Powassan virus, which is endemic in Asia and North America. There is a significant controversy regarding species assignment in the tick-borne encephalitis virus complex due to the complex phylogenetic, serological, ecological, and pathogenetic properties of viruses. Comparing the rate of non-synonymous to synonymous substitutions (dN/dS) over the course of tick-borne orthoflavivirus diversification suggests that there is a very strong stabilizing selection (Nei-Gojobori dN/dS < 0.1) among tick-borne orthoflaviviruses that differ by less than 13.5% amino acid/21.4% nucleotide sequences, and discretely more rapid accumulation of non-synonymous substitutions (dN/dS > 0.13) among more divergent viruses that belong to distinct species. This pattern was similarly observed in genome regions encoding structural (E) and non-structural (NS3) proteins. Below this distance threshold, viruses appear fit and strongly tied to their ecological niche, whereas above the threshold, a greater degree of adaptation appears necessary. This species criterion suggests that all subtypes of TBEV, all related ovine/caprine encephalomyelitis viruses, and Omsk hemorrhagic fever virus (OHFV) together correspond to a single species. Within this species, viruses make up 11 subtypes that are reliably segregated by a 10% nucleotide distance cut-off suggested earlier for TBEV. The same 10% subtype cut-off suggests that Powassan virus includes two subtypes, Powassan and Deer Tick virus.

## 1. Introduction

Orthoflaviviruses (genus *Orthoflavivirus*) (genus *Orthoflavivirus*) are a group of enveloped viruses with a positive-sense RNA genome of 9–11 Kb. Most orthoflaviviruses are arthropod-borne, transmitted by mosquitoes or ticks. They include such human pathogens as Dengue, yellow fever, West Nile, Zika viruses (all vectored by mosquitoes) and tick-borne encephalitis virus (TBEV), Kiassanur forest disease virus, etc. Mosquito-borne orthoflaviviruses (MBFVs) and tick-borne orthoflaviviruses (TBFVs) make up two distinct phylogenetic groups, while for yet another group of orthoflaviviruses vector information is not known [1,2,3]. In addition, there is a large and constantly expanding group of insect-specific orthoflaviviruses. Some of these viruses group with MBFV, while others represent a separate monophyletic phylogenetic group [4].

The taxonomic classification of orthoflaviviruses stems from early studies conducted in the first half of the 20th century. Species assignment has been relatively straightforward for MBFVs, because they cause well-defined clinical presentations, and because studies done in the second half of the 20th century confirmed their distinction in serological tests [5]. Genome-based orthoflavivirus taxonomy was suggested over 20 years ago, and generally corresponds to the disease and serology-based taxonomy [6]. 

Higher-level taxonomic relationships among the orthoflaviviruses were discussed in the context of vector-host adaptation [4,7]. At the same time, the concept of species and the process of speciation are not clearly defined, to the best of our knowledge [8,9,10,11]. 

TBFVs consist of two major clades: tick-borne mammalian and avian viruses. In addition, the third clade of TBFV consists exclusively of the Kadam virus, which was isolated from hard ticks sampled from mammals [12]. The species definition of TBFVs remains a matter of discussion. The paragon TBFV, tick-borne encephalitis virus, includes three classical subtypes termed Far-Eastern, Siberian, and European (TBEV-FE, SIB, and EUR) [13], and four uncommon subtypes, Baikal-1 (Bkl1), Baikal-2 (Bkl2), Ob’, and Himalayan (Him) [14,15,16,17], which are assigned to species *Orthoflavivirus encephalitidis* [18,19]. It should be noted that the TBEV-EUR subtype is phylogenetically closer to the ovine Louping Ill virus (LIV) than to other TBEV subtypes. This contradicts the basic principles of taxonomy, which require a taxon to be monophyletic [20]. In addition, the taxonomic position of Spanish and Turkish sheep encephalomyelitis viruses (SSEV and TSEV), as well as Spanish goat and Greek goat encephalitis viruses (SGEV and GGEV), remains uncertain. This is further complicated by poor serological distinction and significant cross-neutralization between these viruses [5] and lack of correspondence between clinical presentations and phylogenetic grouping. For example, unlike TBEV-EUR, other European orthoflaviviruses, such as LIV, SSEV, and GGEV, do not cause disease in humans, while Omsk hemorrhagic fever virus (OHFV), which causes a distinct disease, is phylogenetically closer to TBEV than the naturally attenuated Langat virus (LGTV), which is so similar to TBEV serologically that it has been proposed as a live TBEV vaccine [21,22,23]. Although the Langat virus TBEV vaccine was rejected due to an unacceptable level of side effects (35 cases of severe neurological complications out of more than 640,000 recipients), the incidence of TBE decreased by 18.4 times among the recipients, which was close to the effectiveness of modern inactivated TBE vaccines.

In the most recent ICTV report, LIV, GGEV, TSEV, SGEV, and SSEV were combined into one species, *Orthoflavivirus loupingi* [18,19]. These viruses are not phylogenetically distinct and make up two groups (LIV-SGEV-SSEV and GGEV-TSEV), which are interspersed with two TBEV clusters. In an attempt to resolve this contradiction, Bondaryuk et al. suggested assigning the TBEV-EUR subtype to one species, the remaining TBEV subtypes to another species, while classifying LIV as a third species and TSEV and GGEV as a fourth [24]. An earlier study also acknowledged this controversy but suggested combining TBEV and LIV into a single species, and this recommendation can be seen as the mainstream view in current taxonomy [12]. Both of these suggestions were supported phylogenetically, but the first one used geographic and mammalian host data as additional criteria, while the latter combined viruses that share the same tick vector into one large species. OHFV forms a sister clade to TBEV and is currently assigned to species *Orthoflavivirus omskense*.

Recently, we have proposed a quantitative criterion of 10% nucleotide sequence difference in ORF that unambiguously divided TBEVs into seven subtypes [14]. This paper aims at finding similar criteria for TBFVs and the underlying evolutionary patterns that could assist in rational species assignment and suggest mechanisms that shape their evolution.

## 2. Materials and Methods

All sequences available in GenBank as of May 2024 were retrieved using Entrez Direct [25]. For the creation of the TBFV and MBFV datasets, taxa with the following taxIDs were used (Table 1).

It should be noted that there were certain contradictions in the NCBI taxonomy. For example, Kumlinge and Negishi viruses, representatives of TBEV, were deposited to GenBank as separate taxons. Similarly, KOUV, a representative of WNV, was deposited as a separate taxon.

Sequences shorter than 10,000 nucleotides were omitted. All sequences that differed from any other sequence in the dataset by less than 2% of the nucleotide sequence were also excluded using in-house Python script. In addition, sequences that showed traces of a non-natural origin (n = 28) were removed (described in detail in the Section 3). Only protein coding regions of the genomes were used for further analysis.

The final MBFV and TBFV datasets contained 317 and 112 complete open reading frames (ORFs), respectively. The nucleotide and amino acid alignments for the complete ORF and fragments encoding E and NS3 genes were created using MEGA [26] and MAFFT [27]. Codon-based alignments were created using PAL2NAL [28]. Uncorrected pairwise distances for nucleotide and amino acid sequences were calculated and visualized in the R environment using ape [29], phangorn [30], scales [31], gdata [32], and ggplot2 [33] packages.

The pairwise dN/dS ratio was calculated using Nei-Gojobori method [34] implemented in in-house Python script and further visualized in the R environment. The dN/dS ratio was also calculated using the Yang–Nielsen method [35] implemented in PAML program v. 4.10.7 [36,37].

For the clarification of phylogenetic relationships, genomes that differed from any other sequence in the dataset by less than 10% of the nucleotide sequence were omitted. These reduced alignments were used for the construction of the phylogenetic trees for amino acid and nucleotide sequences using the IQ-TREE program [38] with 1000 pseudo-replicates [39] and the best-fit model of nucleotide and amino acid substitution [40]. The dN/dS cladogram was built using the neighbor-joining algorithm [41] based on the pairwise dN/dS ratio between sequences using in-house script in the R environment. 

Alignments and scripts are available at https://github.com/AndreiDeviatkin/repo/blob/main/Selection_pressure_profile_suggests_species_criteria_among_tick-borne_flaviviruses/ (accessed on 26 September 2024).

## 3. Results

The sequence dataset included all non-identical (over 2% nucleotide sequence distance) TBFV genomes and, as a reference set of MBFVs, all non-identical WNV, JEV, USUV, YAOV, MVEV, and DENV genomes. The initial alignment included sequences which markedly fell out of the general pattern of correspondence between pairwise amino acid (aa) and nucleotide (nt) distances (Figure S1). Most of these sequences were identified as vaccine candidate viruses with artificially altered codon usage, or contained obvious errors (such as frameshift mutations). After removing these sequences, the final dataset consisted of 429 viruses (112 tick-borne, 77 WNV-like, and 240 DENV). 

**Figure 1:**
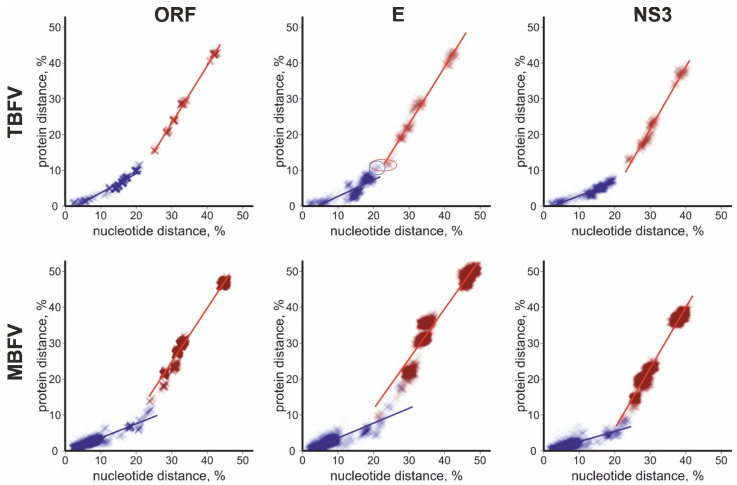
Correspondence between pairwise uncorrected nucleotide and amino acid distances in full ORF, E, and NS3 genome fragments for all tick-borne and reference mosquito-borne (DENV and WNV complex) orthoflaviviruses. Each dot corresponds to a pair of genomic sequences in a dataset. Color indicates distances within species (blue, in the case of TBEV, including OHFV) and between species (red) in the complete ORF, E, and NS3 for TBFV and MBFV. Linear trend lines for inter- and intraspecies distances are shown. Red circle, pairwise distances of LGTV vs. TBEV complex; blue circle, OHFV vs. TBEV complex.

To test the dataset for potential sequence distance cut-offs that can distinguish species, correspondence of pairwise nucleotide and amino acid distances was plotted for TBFVs and MBFVs using complete coding sequences and sequences of the major structural protein E and non-structural protein NS3 (Figure 1). These genomic regions are commonly used to explore genetic relations and taxonomy of orthoflaviviruses and represent proteins with hypothetically distinct evolutionary pressures. Also, previous studies reported incongruence between phylogenetic grouping of TBFV in E and NS3, but only minor difference between NS3 and NS5 [12]. 

In TBFVs, there was a clear gap between intra- and interspecies distance pairs at 21.4–24.6% of nucleotide and 13.5–16.7% amino acid sequences of ORF (Figure 1). This gap was also seen in NS3 at 20.1–22.8% nucleotide and 10.2–13.1% amino acid sequence distances, respectively. In the genome region encoding E protein, intra- and interspecies amino acid distances overlapped, while a narrow gap remained between intra- and interspecies nucleotide distances at 22.0–22.8%. The overlap (Figure 1, red circle) was made up by distances between LGTV and viruses of the TBEV complex. According to these sequence-based cut-offs, OHFV, LIV, GGEV, SGEV, SSEV, and TSEV were members of the TBEV species; therefore, this and other figures were colored accordingly. In other cases, the species designation corresponded to the latest ICTV taxonomy release [18]. In MBFVs (even in the reduced dataset used here), both nucleotide and amino acid intra- and interspecies distances overlapped in full ORF, E, and NS3. Therefore, a universal distance-based criterion was not obvious for MBFVs. 

**Figure 2:**
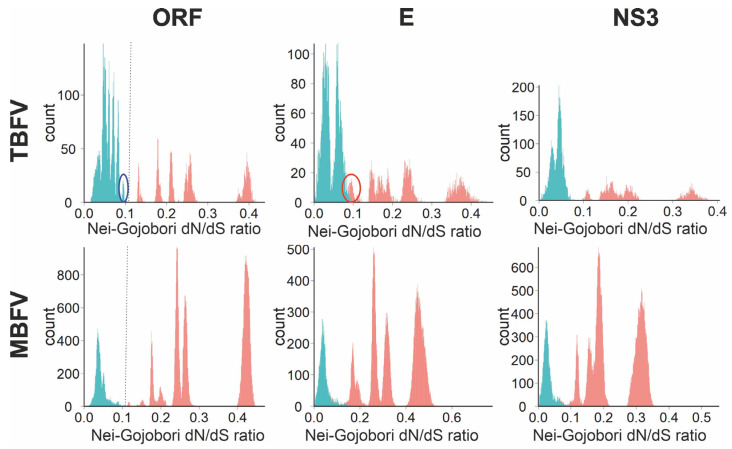
Distribution of pairwise dN/dS ratios among orthoflaviviruses. Cyan, pairs that include viruses of the same species; red, pairs of viruses of distinct species. Dotted lines indicate suggestive cut-offs that distinguish species. Blue circle, OHFV vs. other TBEV complex viruses. Red circle, LGTV vs. TBEV complex viruses.

Similar viruses (over about 75–80% nucleotide sequence identity, corresponding to a species cut-off) featured relatively fewer amino acid substitutions than more divergent ones. For example, #AB507800 (OHFV) and #LC017692 (TBEV, Siberian subtype) differed in 19.7% of the nucleotides and 9.5% of the amino acids of their ORFs, while #AB507800 (OHFV) and #DQ235148 (TYUV) differed in 42.4% of the nucleotides and 42.9% of the amino acids of their ORFs. Many viruses with nucleotide sequence identity over 90–94% had identical or almost identical amino acid sequences. For example, #KJ922514 (TBEV, European subtype) and #MG589937 (TBEV, European subtype) differed in 2.2% of the nucleotides and 0.5% of the amino acids of their ORFs. This pattern was seen in both TBFVs and MBFVs, and similarly in genome parts encoding structural and non-structural proteins. In TBFVs, distinct trends of evolution were clearer in the NS3 encoding region than in E, where a group of pairwise distances of OHFV vs. TBEV/LIV/TGEV (Figure 1, blue circle) deviated from this trend and showed relatively more amino acid substitutions than within TBEV vs. LIV/TGEV. Trend lines of amino acid/nucleotide substitution ratios suggested distinct evolutionary patterns within and between species—an abrupt increase in non-synonymous substitutions and a shift of the ratio between synonymous and non-synonymous substitutions within and between species. To further explore this trend, a distribution of non-synonymous to synonymous substitutions (dN/dS) ratios between viruses of the same and different species was plotted (Figure 2). 

In TBFVs, all intraspecies Nei-Gojobori dN/dS ratios were below 0.103 for ORF and below 0.08 for NS3, while interspecies ratios were above 0.128 and 0.098, respectively. For E protein, intra- and interspecies dN/dS ratios overlapped, and the overlapping interspecies dN/dS ratios (Figure 2, red circle) included LGTV and viruses of the TBEV complex. It is noteworthy that dN/dS ratios between OHFV and TBEV/LIV clearly corresponded to intraspecies distances, although this was found in the higher range of the intraspecies peak (Figure 2, blue circle). In MBFV, intraspecies dN/dS ratios were distinct in the whole ORF and allowed unambiguous discrimination of species at a cut-off of 0.10, at least within the reduced reference dataset used here. In E and NS3, intra- and interspecies dN/dS ratios made up distinct peaks, but they overlapped. This profile corresponded to a better separation of MBFV species in ORF than in NS3 or E by genetic distances (Figure 1). 

It is also noteworthy that a clear distinction between dN/dS ratios within and between species was observed with a simple Nei-Gojobori algorithm. A more advanced Yang–Nielsen dN/dS method yielded the same overall pattern (lower dN/dS within species); however, the intra- and interspecies dN/dS ratios partially overlapped (Figure S2). Although more advanced methods may better reflect evolutionary relations of viruses, a more straightforward approach was better suited for the utilitarian purpose of distinguishing species. 

**Figure 3:**
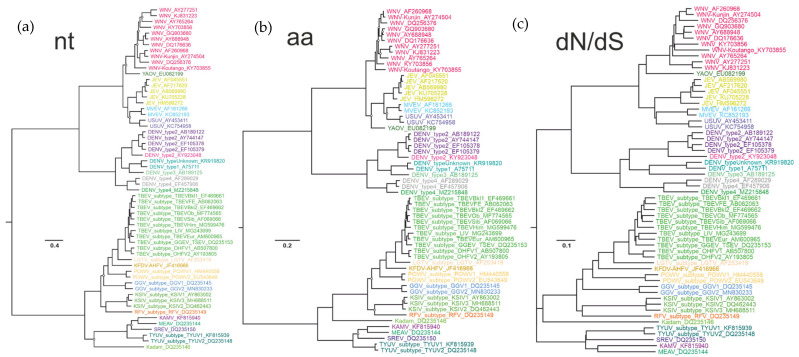
(**a**,**b**) full ORF nucleotide and amino acid sequence-based ML phylogenetic trees for TBFVs and reference MBFVs. Genomes with >90% nucleotide sequence identity were omitted. Tip colors correspond to species suggested by the 0.11 dN/dS cut-off. Hollow circles indicate tree nodes with <95% bootstrap support. (**c**) cladogram for TBFVs and MBFVs based on the dN/dS ratios (Nei-Gojobori); no bootstrap test was done for the cladogram.

In orthoflaviviruses, the degree of stabilizing pressure (evident as lower dN/dS ratios) corresponded to the relatedness of the viruses. To further illustrate this, phylogenetic trees inferred from using nucleotide and amino acid sequences (Figure 3, left and central panel) were compared to a cladogram built using the pairwise Nei-Gojobori dN/dS matrix and neighbor-joining algorithm (Figure 3, right panel). The dN/dS cladogram corresponded to phylogenetic trees, confirming the relation between the degree of selection pressure and phylogenetic relatedness. 

**Figure 4:**
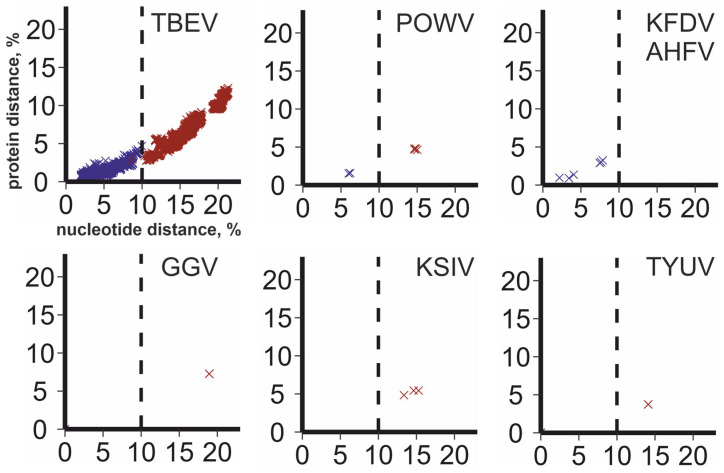
Correspondence between uncorrected pairwise nucleotide and amino acid distances in full ORF for TBEV, POWV, KFDV, AHFV, GGV, KSIV, and TYUV. Each dot corresponds to a pair of genomic sequences in a dataset. Color indicates distances within subtype (blue) and between subtypes (red). Dashed line indicates the suggested 10% nucleotide sequence cut-off for subtypes.

Phylogenetic trees (Figure 3) were inferred using a dataset created using a 10% ORF nucleotide sequence cut-off that was suggested previously for TBEV subtypes [14], and included one member of each of the seven TBEV subtypes. In addition, four subtypes could be annotated within the TBEV species according to the 10% nucleotide distance cut-off criteria: (1) LIV, SGEV, SSEV; (2) GGEV, TSEV; (3) OHFV subtype 1; (4) OHFV subtype 2. Furthermore, two prospective subtypes of POWV, GGV, and TYUV, and three subtypes of KSIV were identified within the corresponding virus species. We suggested that the 10% nucleotide sequence cut-off may be universally applicable to TBFV subtypes beyond TBEV sensu stricto. To illustrate this segregation, the distribution of nucleotide distances among the selected TBFV (data from Figure 1) was colored according to the prospective subtypes (Figure 4). All subtypes discussed above were reliably distinguished by a 10% nucleotide distance cut-off within the complete ORF. There was one exclusion from this rule. Genbank entry #OP292291 (representative of one OHFV subtype) had 8.7% nucleotide sequence difference from #MT354616 (representative of another OHFV subtype). The sequence #OP292291 turned out to be mosaic relative to other OHFV genomes (Figure S3); however, it was not possible to conclude if this was an unusual natural recombination event or a sequencing error.

## 4. Discussion

The concept of species in biology is generally tied to sexual reproduction, and its application in virology is not always obvious. Species that were assigned to viruses are often based on historical considerations or clinical presentations, which may contradict the phylogenetic evidence. Moreover, molecular distances and phylogenetic groupings are not comprehensive, due to sampling bias (including anthropocentric virus sampling), founder effects, and random bottlenecks. It is therefore desirable to understand biological phenomena that could explain why viruses do not occupy all possible sequence space evenly. 

Early studies have identified very high mutation and substitution rates among RNA viruses. However, the concept of rapidly changing RNA viruses contradicts findings of integrated viral sequences in insect genomes, suggesting that viruses have remained stable over millions of years. It has been proposed that viruses are “prisoners” of their ecological niches and cannot drift beyond the boundaries that ensure their sustainable survival in their historical settings [42]. The extreme stabilization of orthoflavivirus genomes within a prospective species (below approximately 22% nucleotide distance cut-off) with dN/dS ratios below 0.1 is compatible with this hypothetical niche adaptation. Similar profiles of such adaptation in TBFVs and MBFVs could be coincidental, but they could also suggest that boundaries of a niche (and, correspondingly, species) could represent an ancient trait of orthoflaviviruses. It was also unexpected to observe comparable substitution patterns and, correspondingly, niche boundaries in structural and non-structural proteins. In many viruses, structural proteins are much more variable, largely owing to pressure from the adaptive immune system. Obviously, this was not the case in either TBFVs or MBFVs. This suggests that the E protein is not subject to significant pressure from the mammalian immune system, the implication of this being a low probability of vaccine escape among orthoflaviviruses. This has been observed for nearly a century in the YFV vaccine and the TBEV vaccine, and it can be suggested as a general feature of orthoflaviviruses. Additionally, this stability implies that both structural and non-structural proteins of orthoflaviviruses are suitable for taxonomic classification purposes, although NS3 provided a better distinction of species. 

In the era of extensive virus discovery, taxonomic assignment of newly identified viruses could benefit from straightforward sequence distance criteria. So far, it has been difficult to infer universal distance cut-offs in orthoflaviviruses. A seminal study suggested cut-off distances for species demarcation at 0.09 based on the complete ORF amino acid sequences and at 0.063 based on the complete NS3 protein [12]. However, the distribution of genetic distances among the currently known orthoflaviviruses (Figure 1) indicates that this cut-off was not universal. A nucleotide distance cut-off of 10% was superior to amino acid sequence distance-based criteria to distinguish TBEV subspecies [14]. Similarly, nucleotide sequence distances within and between putative species could be better distinguished (had a wider “white zone” without pairwise distances) than amino acid sequences (Figure 1), but only for TBF. A better distinction of species was seen for trends of correspondence between nucleotide and amino acid substitutions (Figure 1) and, correspondingly, for dN/dS ratios (Figure 2). This hypothetically corresponds to a shift in pressure mode that shaped viruses at different levels of their diversification (stabilizing vs. relatively more modifying selection) and thus makes more sense in terms of evolutionary mechanisms than sequence distances, which are more likely to be subject to various sources of bias. The dN/dS cladogram corresponded to phylogenetic grouping (Figure 3), concordant with the observation that the rate of non-synonymous substitution increases with genetic distance. 

It is noteworthy that the best distinction of species was observed for uncorrected genetic distances (Figure 1, data not shown for other models of nucleotide distance calculation) and for the most basic method of dN/dS calculation (Figure S1). It may be suggested that more advanced methods compensate for nonlinear trends in accumulation of substitutions, which provides a more accurate estimation of phylogenetic relationships but results in poorer distinctions of taxa, which was the purpose of this study. While it is tempting to suggest dN/dS ratios as an additional taxonomic criterion, their robustness requires further confirmation, especially since not all algorithms provide distinctions among orthoflavivirus species.

Flaviviruses exemplify the difficulty of accommodating historical classification into a modern genome-based taxonomy. It is obvious that TBEV sensu stricto cannot be designated a single species because it is not phylogenetically distinct from LIV and TGEV (see above). Two major approaches to resolve this controversy are to create larger species, which include TBEV and LIV/TGEV [12], or smaller species corresponding to specific viruses [24,43]. The first approach appears more compatible with the genetic relations of the MBFVs, which feature much higher genetic distances within taxa of the same level. For example, DENV of the same serotype may differ by up to 18.3% of nucleotide sequence [44], while subtypes of TBEV may be distinguished by a 10% nucleotide distance threshold [14]. Pairwise nucleotide sequence distances in our dataset were up to 34.4% between the most divergent members of the species DENV, #KJ755855 and #JQ922559 (based on the ORF nucleotide sequences), 17.9% within the TBEV+LIV species (#KT001073 and #KJ495985) suggested by the large species approach [12], and 8.82% within the potential TBEV-Eur species (#ON502378 and #KF991107) suggested by a small species approach [24]. Therefore, distance criteria, both amino acid and nucleotide, favor the large species classification (Figure 1). However, in the absence of mechanistic explanation, any distance criterion would not be robust. Comparing the selection pressure between TBFVs and MBFVs showed remarkably similar patterns. Extreme negative selection (dN/dS ratios below 0.11) was evident up to nucleotide distances of approximately 22%. Above this threshold, accumulation of non-synonymous substitutions became more balanced, yet remained below an arbitrary neutral selection threshold of 1 (Figure 2). Therefore, species were shaped rather by conservation of amino acid sequence than by diversification, and there was a shift in strength of pressure at the prospective species boundary. According to this criterion, hypothetically based on evolutionary mechanisms, TBEV species include seven subtypes of TBEV, LIV/SSEV/SGEV, TSEV/GGEV, and two OHFV subtypes.

Within this suggestive large species, viruses could be distinguished by the same 10% nucleotide sequence cut-off in the whole ORF (Figure 4) that was suggested previously for TBEV sensu stricto [14]. This segregation was concordant with the phylogenetic grouping observed previously [12] and suggests that LIV, SGEV, and SSEV are the same subtypes, TSEV and GGEV are another one, and OHFV includes two subtypes. The same 10% cut-off appeared universal for all TBFV. KFDV and AHFV fell into one subtype. Karshi virus included three subtypes, while Powassan, Gadgets Gully, and Tyuleniy viruses made up two subtypes each. There are well known differences in the geographic distribution and host range of TBEV and POWV subtypes. Therefore, a comparable degree of variation may be anticipated between subtypes of GGV and KSIV. On the other hand, the suggestion to assign two variants of GGV to two distinct species [24] was not compatible with the taxonomic relations among other TBFVs. From a practical point of view, it is known that the TBEV vaccine protects against heterologous subtypes [45,46], therefore, comparable properties might be expected for vaccines against less common TBFVs [46,47].

## 5. Conclusions

**Taxonomic proposal.** Evolutionary patterns among TBFVs suggest that one species, suggestively *Orthoflavivirus eurasiaticus*, delimited by a nucleotide sequence difference of 23%, amino acid sequence difference of 15%, and dN/dS ratio <0.11 in the polyprotein, should include eleven subtypes (or subspecies), namely seven subtypes of TBEV (TBEV-FE, Sib, Eur, Bkl1, Bkl2, Ob and Him), LIV (including SSEV and SGEV), TSEV (including GGEV), and two subtypes of OHFV (OHFV1 and OHFV2) delimited by a 10% nucleotide sequence cut-off over the whole ORF, in addition to the host range and pathogenic profile.

## Figures and Tables

**Table 1:** Taxa of TBFVs and MBFVs used in the analysis.

Virus Name, Abbreviation	taxID	Virus Species According to Current ICTV Taxonomy
TBFV		
Alkhurma hemorrhagic fever virus, AHFV	txid172148	*Orthoflavivirus kyasanurense*
Deer tick virus, DTV	txid58535	*Orthoflavivirus powassanense*
Gadgets Gully virus, GGV	txid64307	*Orthoflavivirus gadgetsense*
Greek goat encephalitis virus, GGEV	txid41406	*Orthoflavivirus loupingi*
Kadam virus, KADV	txid64310	*Orthoflavivirus kadamense*
Kama virus, KAMV	txid1456752	unclassified
Karshi virus, KSIV	txid64287	unclassified
Kumlinge virus	txid11092	*Orthoflavivirus encephalitidis*
Kyasanur Forest disease, KFDV	txid33743	*Orthoflavivirus kyasanurense*
Langat virus, LGTV	txid11085	*Orthoflavivirus langatense*
Louping Ill virus, LIV	txid11086	*Orthoflavivirus loupingi*
Meaban virus, MEAV	txid35279	*Orthoflavivirus meabanense*
Negishi virus	txid11094	*Orthoflavivirus encephalitidis*
Omsk hemorrhagic fever virus, OHFV	txid12542	*Orthoflavivirus omskense*
Powassan virus, POWV	txid11083	*Orthoflavivirus powassanense*
Royal Farm virus, RFV	txid64288	*Orthoflavivirus royalense*
Saumarez Reef virus, SREV	txid40012	*Orthoflavivirus saumarezense*
Spanish goat encephalitis virus, SGEV	txid1691889	*Orthoflavivirus loupingi*
Spanish sheep encephalitis virus, SSEV	txid41408	*Orthoflavivirus loupingi*
Tick-borne encephalitis virus, TBEV	txid11084	*Orthoflavivirus encephalitidis*
Turkish sheep encephalomyelitis virus, TSEV	txid47300	*Orthoflavivirus loupingi*
Tyuleniy virus, TYUV	txid40004	*Orthoflavivirus tyuleniyense*
**MBFV**		
Alfuy virus, ALFV	txid11079	*Orthoflavivirus murrayense*
Dengue virus, DENV	txid12637	*Orthoflavivirus denguei*
Japanese encephalitis virus, JEV	txid11072	*Orthoflavivirus japonicum*
Koutango virus, KOUV	txid44025	*Orthoflavivirus koutangoense*
Murray Valley encephalitis virus, MVEV	txid11079	*Orthoflavivirus murrayense*
Usutu virus, USUV	txid64286	*Orthoflavivirus usutuense*
West Nile Virus, WNV	txid11082	*Orthoflavivirus nilense*
Yaounde virus, YAOV	txid64319	*Orthoflavivirus yaoundeense*

## Data Availability

Datasets and scripts used in the study are available at https://github.com/AndreiDeviatkin/repo/blob/main/Selection_pressure_profile_suggests_species_criteria_among_tick-borne_flaviviruses/.

## Supplementary Materials

The following supporting information can be downloaded at: https://www.mdpi.com/article/10.3390/v16101554/s1, Figure S1: Correspondence between uncorrected pairwise nucleotide and amino acid distances in full ORF for all tick-borne and reference mosquito-borne (DENV and WNV complex) orthoflaviviruses. Each dot corresponds to a pair of genomic sequences in a dataset. Unfiltered datasets (right column) included artificial and erroneous sequences; Figure S2: Distribution of pairwise Yang-Nielsen dN/dS ratios among orthoflaviviruses. Cyan, pairs that include viruses of the same species; red, pairs of viruses of distinct species.; Figure S3: Similarity plot for a recombinant OHFV (Genbank entry #OP292291) based on OHFV sequences from the complete dataset (window = 500 nt, step = 20 nt). The x-axis indicates the genomic position (sliding window center); the y-axis shows the percent identity between the query sequence and six other OHFV genomes. Analysis was done using SimPlot 3.5.1 [48].
