# Supplementary material for: Selection Pressure Profile Suggests Species Criteria among Tick-Borne Orthoflaviviruses
Source: Viruses. 2024 Sep 30;16(10):1554. doi: 10.3390/v16101554 (PMC11512272; doi:10.3390/v16101554)
Supplement: Supplementary file 1 [file viruses-16-01554-s001.zip › viruses-3203130-supplementary.pdf]

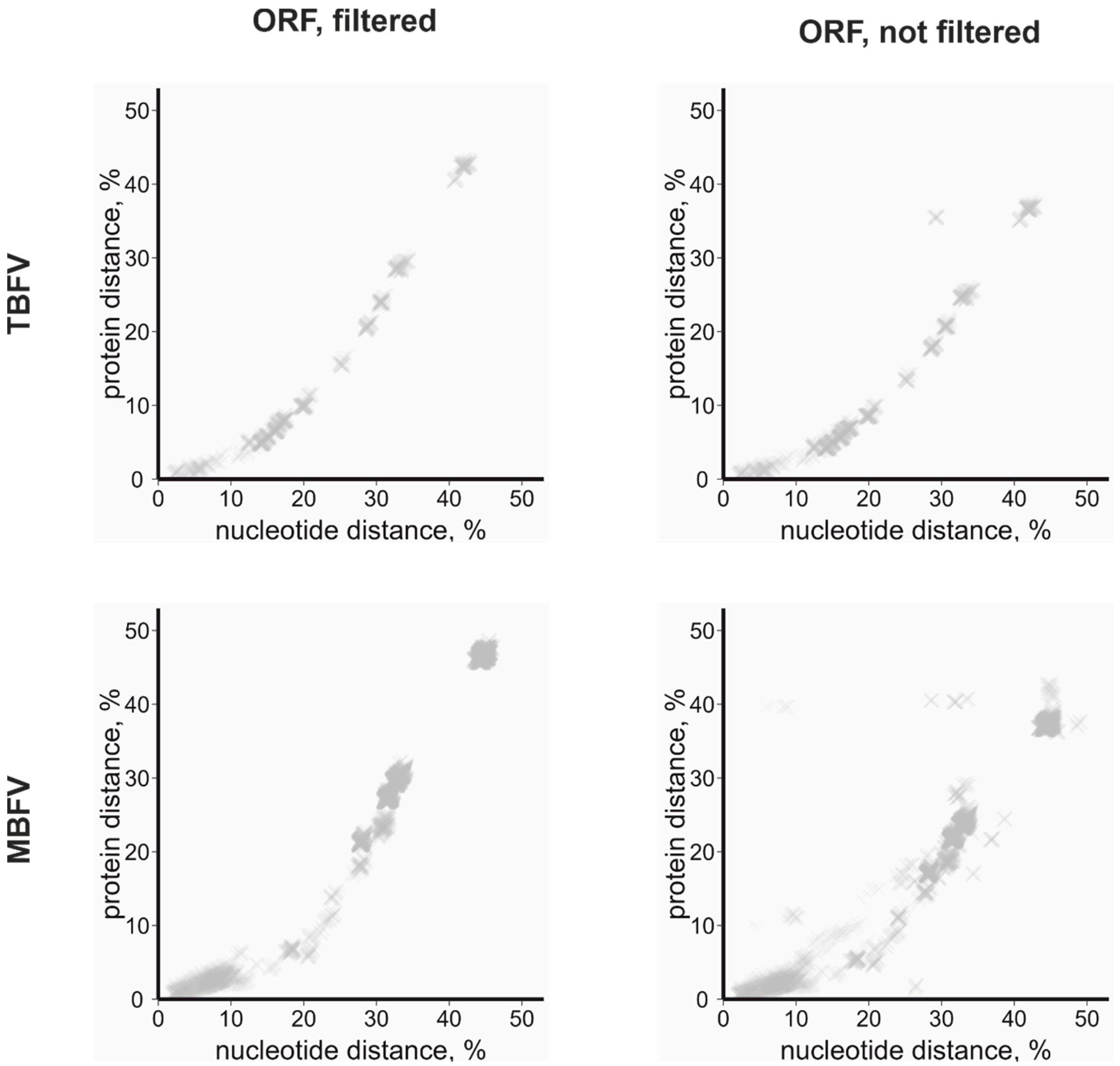

Figure S1. Correspondence between uncorrected pairwise nucleotide and amino acid distances in full ORF for all tick-borne and reference mosquito-borne (DENV and WNV complex) orthoflaviruses. Each dot corresponds to a pair of genomic sequences in a dataset. Unfiltered datasets (right column) included artificial and erroneous sequences.

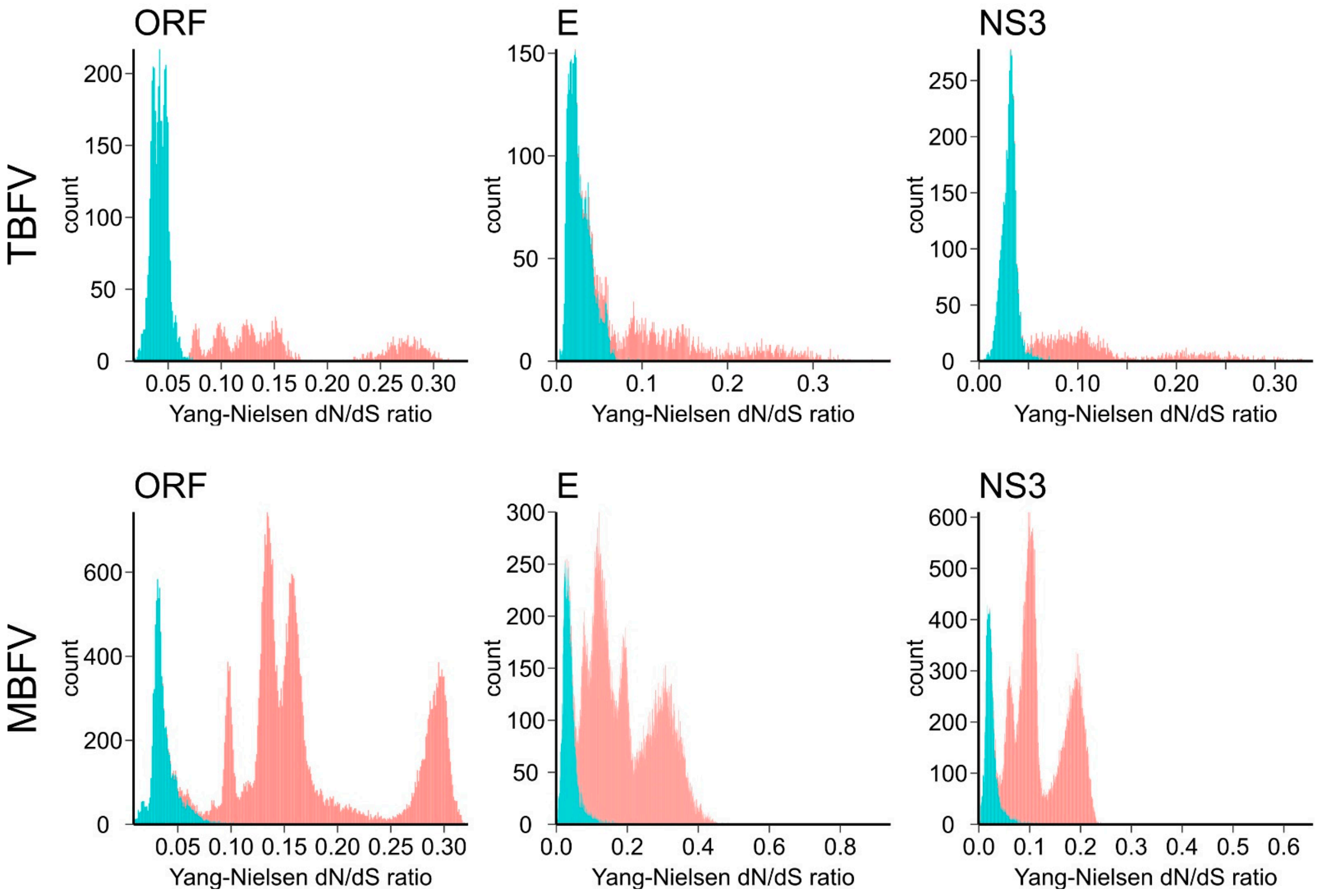

Figure S2. Distribution of pairwise Yang-Nielsen dN/dS ratios among orthoflaviruses. Cyan, pairs that include viruses of the same species; red, pairs of viruses of distinct species.

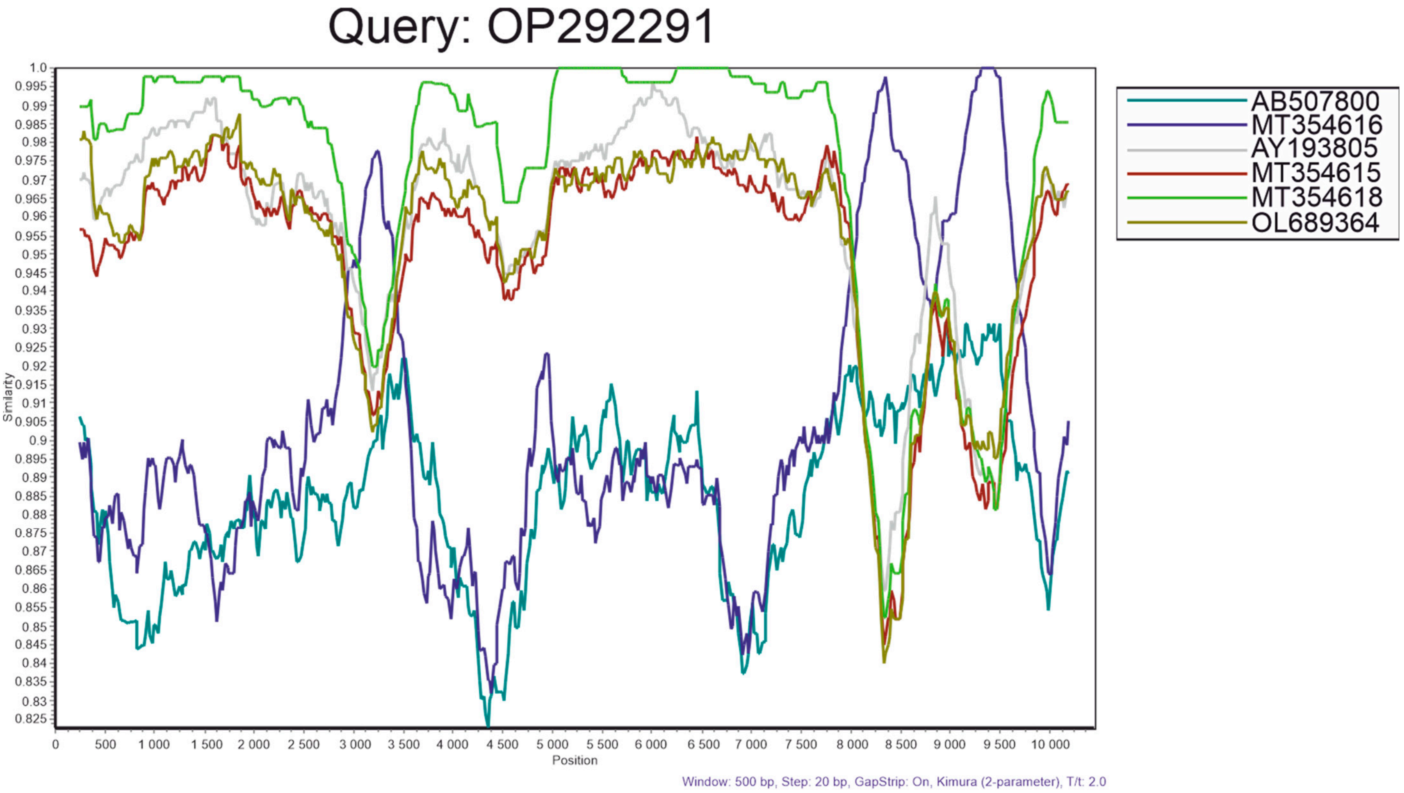

Figure S3. Similarity plot for a recombinant OHFV (Genbank entry #OP292291) based on OHFV sequences from the complete dataset (window = 500 nt, step = 20 nt). The x-axis indicates the genomic position (sliding window center); the y-axis shows the percent identity between the query sequence and six other OHFV genomes. Analysis was done using SimPlot 3.5.1 [48].
